# First record of the rare genus *Typhrasa* (Psathyrellaceae, Agaricales) from China with description of two new species

**DOI:** 10.3897/mycokeys.79.63700

**Published:** 2021-04-23

**Authors:** Sheng-Nan Wang, Ya-Ping Hu*, Jun-Liang Chen, Liang-Liang Qi, Hui Zeng, Hui Ding, Guang-Hua Huo, Lin-Ping Zhang, Fu-Sheng Chen, Jun-Qing Yan

**Affiliations:** 1 Jiangxi Key Laboratory for Conservation and Utilization of Fungal Resource; Key Laboratory of State Forestry Administration on Forest Ecosystem Protection and Restoration of Poyang Lake Watershed, Jiangxi Agricultural University, Nanchang, Jiangxi 330045, China Jiangxi Agricultural University Nanchang China; 2 Institute of Environmental Sciences, Ministry of Ecology and Environment/ State Environmental Protection Scient-ific Observation and Research Station for Ecological Environment of Wuyi Mountains 8 Jiangwangmiao Street, Nanjing 210042, China Institute of Environmental Sciences Nanjing China; 3 Science and Technology Research Center of Edible Fungi, Qingyuan, Zhejiang, 323800, China Science and Technology Research Center of Edible Fungi Qingyuan China; 4 Microbiology Research Institute, Guangxi Academy of Agriculture Sciences, Nanning, 530007, China Microbiology Research Institute, Guangxi Academy of Agriculture Sciences Nanning China; 5 Institute of Edible Fungi, Fujian Academy of Agricultural Sciences; National and Local Joint Engineering Research Center for Breeding & Cultivation of Features Edible Fungi, Fuzhou 350014, China Institute of Edible Fungi, Fujian Academy of Agricultural Sciences Fuzhou China

**Keywords:** Basidiomycota, macromycetes, morphology, phylogenetic analysis, taxonomy

## Abstract

*Typhrasa* is a rare genus that comprises two species and that has previously been reported only from Europe and North America. The present study expands the geographical scope of the genus by describing two new species – *T. polycystis* and *T. rugocephala* – from subtropical China. The new species are supported by morphological characteristics and phylogenetic analyses (ITS, LSU and *tef-1α*). The new species have very similar morphological characteristics and are 98% similar in their ITS region. However, *T. rugocephala* has two types of long gills at the same time, rarely fusiform pleurocystidia with rostrum. Detailed descriptions, colour photos, illustrations and a key to related species are presented in this paper.

## Introduction

The genus *Typhrasa* Örstadius & E. Larss. was established in 2015. It is characterised by a hygrophanous cap, crowded gills with white edge, small-to-medium-sized spores, large hymenial cystidia with intracellular oily drops or globules and a hymeniderm or paraderm pileipellis (Örstadius et al. 2015). The genus includes two species, *T. gossypina* (Bull.) Örstadius & E. Larss. and *T. nanispora* Örstadius, Hauskn. & E. Larss., the former being previously reported occasionally from some countries in Europe, North America and Asia within the genus *Psathyrella* (Fr.) Quél (Smith 1972; Kits van Waveren 1985; Knudsen and Vesterholt 2012; Örstadius et al. 2015). In addition, *Psathyrella delineata* (Peck) A.H. Sm., *P. canadensis* A.H. Sm. and *P. subtenacipes* A.H. Sm. are also reported to have oily drops in their cystidia (Smith 1972; Kits van Waveren 1985) and seems to be a candidate for *Typhrasa*. However, *P. delineata* and *P. canadensis* were combined into *T. gossypina*, based on the morphology (Örstadius et al. 2015). During investigations in subtropical China during 2018–2020, *Typhrasa* was recorded for the first time in China with two unrecorded species, which were frequently collected. Based on morphological characters and phylogenetic analyses, they are described as new species in this paper.

## Materials and methods

### Morphological studies

Macromorphological characters and habitat details were noted from fresh, young to mature basidiomata (over five basidiomata for each species) in the field. The location of the collection point is marked on the map (Suppl. material 1: Fig. S1). Colour codes are from the Methuen Handbook of Colour (Kornerup and Wanscher 1978). Micromorphological characters were observed with a light microscope (Olympus BX53). Sections from dry specimens were observed in water, 5% aqueous potassium hydroxide (KOH) solution, 10% aqueous ammonia (NH_3_·H_2_O) solution and Melzer’s Reagent, separately. More than fifty basidiospores, cystidia and basidia in 5% aqueous KOH solution were measured under the microscope. Basidiospore measurements were recorded in front and profile view. The measurements and Q values are given as (a)b–c(d), in which “a” is the lowest value, “b–c” covers a minimum of 90% of the values and “d” is the highest value. “Q” represents the ratio of length to width of a spore (Bas 1969; Ge et al. 2017; Na and Bau 2019). Specimens are deposited in the Herbarium of Fungi, Jiangxi Agricultural University (**HFJAU**) and Herbarium of Mycology, Jilin Agricultural University (**HMJAU**).

### DNA extraction and sequencing

DNA was extracted from dried specimens with the NuClean Plant Genomic DNA kit (CWBIO, China). Three regions (ITS, LSU and *Tef-1α*) were generated for the study, which were amplified with primers ITS1/ITS4 (White et al. 1990), LR0R/LR7 (Hopple and Vilgalys 1999) and EF983F/EF2218R (Örstadius et al. 2015), respectively. PCR was performed using a touchdown programme: 5 min at 95 °C; 1 min at 95 °C; 30 s at 65 °C (add -1 °C per cycle); 1 min at 72 °C; 15 cycles; 1 min at 95 °C; 30 s at 50 °C; 1 min at 72 °C; 20 cycles; and 10 min at 72 °C (Yan and Bau 2018). The DNA sequencing was done by Qing Ke Biotechnology Co. Ltd. (Wuhan City, China).

### Data analyses

The ITS, LSU and *Tef-1α* datasets were assembled following Örstadius et al. (2015) and BLAST in GenBank. Sequences from a total of 24 taxa were analysed using five data partitions (ITS, LSU, Tef 1^st^, Tef 2^nd^ and Tef 3^rd^). The details are presented in Table 1. Sequences were aligned separately in MAFFT v.7 (Katoh and Standley 2013). The best-fit models of nucleotide evolution for ITS, LSU, Tef 1^st^, Tef 2^nd^ and Tef 3^rd^ datasets (GTR+G, GTR+I, SYM, SYM and GTR+G, respectively) were obtained in MrModeltest v.2.3 (Nylander et al. 2008). Phylogenetic analysis was conducted using Bayesian Inference (BI) in MrBayes v.3.2.6 (Ronquist et al. 2012). Gaps were treated as missing data following Örstadius et al. (2015). Four Monte Carlo Markov Chains (MCMC) were run for five million generations, sampling every 100^th^ generation. The first 25% of trees were discarded as burn-in (Ronquist et al. 2012). The sequence alignment is deposited in TreeBASE (http://purl.org/phylo/treebase/phylows/study/TB2:S27860).

**Table 1.** Sequences used in this study. Newly generated sequences are given in bold. Type material is indicated in the column Voucher.

Taxa	Voucher	Locality	ITS	LSU	*tef*-*1*α
*Cystoagaricus hirtosquamulosus*	Ramsholm800927	Finland	KC992945	KC992945	–
*C. olivaceogriseus*	WK 8/15/63-5 (MICH) Type	USA	KC992948	KC992948	–
*C. sylvestris*	LÖ191-92	Sweden	KC992949	KC992949	–
*C. squarrosiceps*	Laessoe44835	Ecuador	KC992950	–	–
*C. strobilomyces*	E. Nagasawa 9740		AY176347	AY176348	–
*Kauffmania larga*	LAS97-054	Sweden	DQ389695	DQ389695	–
*K. larga*	LÖ223-90	Sweden	DQ389694	DQ389694	KJ732824
*Psathyrella delineata*	CCB171	USA	KY744151		
*P. delineata*	TMW02	USA	MF686534		
*P. delineata*	MGW1406	USA	KY777378		
*Typhrasa gossypina*	180524-H08	Korea	MN082538	–	–
*T. gossypina*	BRNM:705622	Austria	AM712293	–	–
*T. gossypina*	BRNM:705609	Czech	AM712292	–	–
*T. gossypina*	WU:25069	Austria	AM712294	–	–
*T. gossypina*	Schumacher024	Germany	KC992946	KC992946	KJ732825
*T. nanispora*	Barta980706 Type	Austria	KC992947	KC992947	–
*T. polycystis*	HFJAU1454 Type	China:Jiangxi	**MW466538**	**MW466544**	**MW475280**
*T. polycystis*	HFJAU1520	China:Fujian	**MW466539**	**MW466545**	**MW475281**
*T. polycystis*	HFJAU1349	China:Jiangxi	**MW466540**	–	–
*T. rugocephala*	HFJAU1467 Type	China:Zhejiang	**MW466541**	**MW466546**	**MW475282**
*T. rugocephala*	HFJAU1455	China:Zhejiang	**MW466542**	**MW466547**	**MW475283**
*T. rugocephala*	HFJAU1476	China:Zhejiang	**MW466543**	**MW466548**	–
**Outgroup**					
*Psathyrella oboensis*	DED 8234 Type	SãoTomé	NR148107	–	–
*P. pertinax*	LO259-91 Neotype	Sweden	DQ389701	DQ389701	KJ732809

## Results

**Figure 1.**
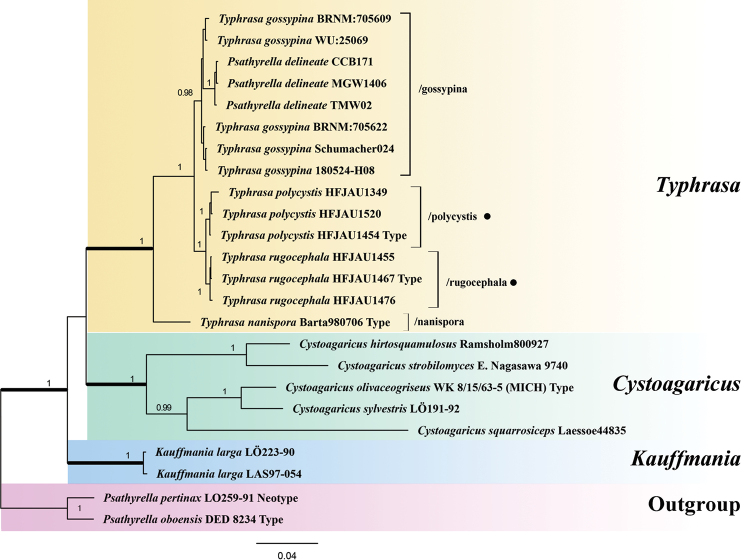
Phylogram generated by Bayesian Inference (BI) analysis, based on sequences of a concatenated dataset from three nuclear markers (ITS, LSU and *tef-1α*) rooted with *Psathyrella* spp. Bayesian posterior probabilities ≥ 0.95 are shown. ● indicates the newly-described species.

Based on the BLAST results of the full length of the ITS region, two new species were found sharing less than 98.0% similarity with the known species of *Typhrasa*, respectively: 97% with *T. gossypina* and 92% with *T. nanispora*. The Bayesian analysis (Figure 1) comprised material from three major genera, all of which are identified as monophyletic with very strong support (BPP = 1), viz. *Typhrasa*, *Cystoagaricus* Singer and *Kauffmania* Örstadius & E. Larss., in agreement with the study published by Örstadius et al. (2015). Our collections of the two new *Typhrasa* species formed a joint, strongly supported clade (BPP = 1) and both new species received strong support as monophyletic (BPP ≥ 0.97). The collections of *T. gossypina* (Bull.) Örstadius & E. Larss. clustered together and appeared as a sister to the group consisting of the two new species.

### Taxonomy

#### Typhrasa polycystis

Taxon classificationFungiAgaricalesPsathyrellaceae

J.Q Yan & S.N. Wang sp. nov.

11360C03-9F5B-50D7-A968-94A3D152A7EA

838482

Fig. 2

##### Diagnosis.

Differs from *Typhrasa gossypina* by its smaller spores (7.1–8.2 × 4.3–5.1 μm).

##### Holotype.

China. Jiulianshan National Nature Reserve, Jiangxi Province, 25 June 2020, Jun-Qing Yan, HFJAU1454.

##### Etymology.

Referring to the characteristics of the pleurocystidia.

##### Description.

Pileus 20–35 mm, extending hemispherically to expanded, plane with or without umbo, surface with slightly ridge-like folds or smooth, hygrophanous, brown (7D6–7C6), pale brown (6B6–6C6) at the margin. Veil distinct, fibrous or fluffy, white (7C1), markedly appendiculate at margin, falling off easily. Context thin and fragile, hygrophanous at pileus, about 3.0 mm at the centre. Gills 4.0–5.0 mm broad, moderately close, pale cinnamon (6C6–6D5) with a white (6C1) edge, adnexed. Stipe 30–45 mm long, 5.0–8.0 mm thick, white (6C1), hollow, pulverulent at apex, with fibrils and fluffy from pellicular veil remnants below, falling off easily.

Spores (6.9)7.1–8.2 × (4.2)4.3–5.1(5.2) μm, Q = (1.4)1.5–1.8(1.9), ellipsoid to oblong-ellipsoid, profile flattened on one side, 4.1–5.0(5.2) μm broad, smooth, reddish-brown (8C5–8C6) in water, yellow-brown (7D6–7D7) in 5% KOH or 10% NH_3_·H_2_O, becoming darker (7E4–7F4) in 5% KOH, germ pore small and indistinct, 1–2 guttulate, inamyloid. Basidia 21–24 × 7.5–8.5 μm, 4-spored, clavate, hyaline. Pleurocystidia 55–81(87) × 11–17(18) μm, variously shaped, often fusiform and lageniform, with rostrum; rarely fusiform with subacute to acute apex or cystiform with rostrum, with one or two large internal oily drops, oily drops colourless and distinct or indistinct in 5% KOH, glassy-yellow (5B6–5B7) and very distinct in Melzer’s Reagent. Cheilocystidia (24)30–54(58) × (9)10–15(16) μm, similar to pleurocystidia, abundant, rarely mixed with pyriform or clavate cells. Trama of gills consisting of parallel hyphae. Pileipellis a 2–3 cells deep layer of subglobose or pyriform cells which are 24–36 μm wide. Veil composed of hyphae 6.5–14.5 μm-broad, thin-walled and fawn (5A2-A3) hyphae in 5% KOH. Clamps present in trama of gills, hyphae of stipe and at the base of the basidia and cystidia.

##### Ecology and distribution.

Saprotrophic, solitary to slightly caespitose on rotten hard wood or humus in mixed forests.

##### Other specimens examined.

China. Jiulianshan National Nature Reserve, Jiangxi Province, 28 May 2018, Guang-Hua Huo, Lin-Ping Zhang, HFJAU1349. Wuyishan National Nature Reserve, Fujian Province, 27.748888°N, 117.7625°E, 761 alt., 12 June 2020, Liangliang Qi, Yupeng Ge, HFJAU1520, HMJAU58461.

**Figure 2.**
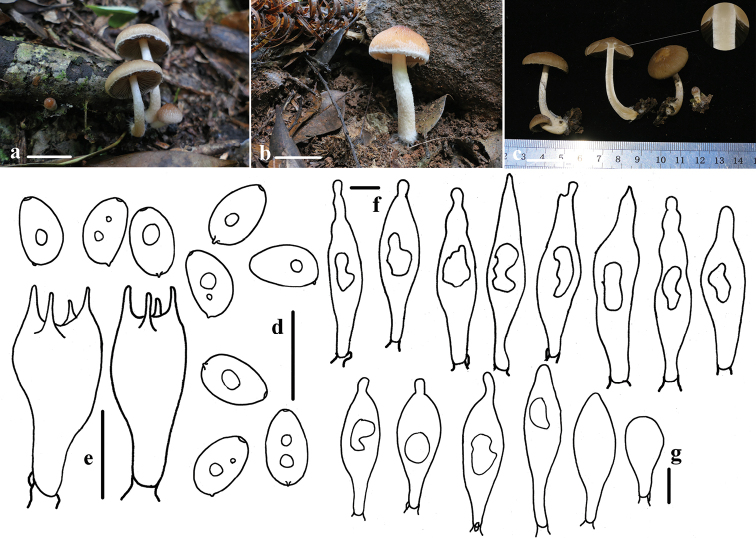
Basidiomata and microscopic features of *Typhrasa polycystis***a–c** basidiomata **d** basidiospores **e** basidia **f** pleurocystidia **g** cheilocystidia. Scale bars: 20 mm (**a–c**); 10 μm (**d–g**).

#### Typhrasa rugocephala

Taxon classificationFungiAgaricalesPsathyrellaceae

J.Q Yan & S.N. Wang sp. nov.

0BF3F3E7-9EE6-5283-9CCB-10FC36A5A2AE

838483

Fig. 3

##### Diagnosis.

Differs from *Typhrasa polycystis* by having two types of long gills and rarely rostrum can be found in fusiform pleurocystidia.

##### Holotype.

China. Baishanzu National Nature Reserve, Zhejiang Province, 27.734233°N, 119.186943°E, 1184 m alt. 28 June 2020, Sheng-Nan Wang, HFJAU1467.

##### Etymology.

Referring to the surface of the pileus.

##### Description.

Pileus 35–55 mm, spreading hemispherically to oblate with a slight umbo, surface with distinct ridge-like folds, hygrophanous, reddish-brown (8E5–8﻿F6), pale brown (6D7–6C7) at the margin, drying tawny (7D6–7E6), striate, sometimes faintly, at margin. Veil distinct, fibrous or fluffy, white (7C1), markedly appendiculate at margin, falling off easily. Context thin and fragile, hygrophanous, about 2.5 mm at the centre. Gills 5.0–7.0 mm broad, moderately close; when young, dirty white (7B1), becoming cinnamon (7C6–7D5) with a white edge (7C1); two types of long gills arranged at intervals: A: adnate to slightly decurrent, B: emarginate- adnexed. Stipe 40–60 mm long, 5.0–10 mm thick, white (7C1), hollow, pulverulent at apex, with fibrils and fluffy from pellicular veil remnants below, falling off easily.

Spores (6.5)6.8–7.9(8.3) × 4.5–5.2(5.4) μm, Q = (1.3)1.4–1.7, ellipsoid to oblong-ellipsoid, profile flattened on one side, 4.1–5.0(5.2) μm broad, smooth, reddish-brown (8C6–8C7) in water, yellow-brown (7D6–7D7) in 5% KOH or 10% NH_3_·H_2_O, becoming darker (7E4–7E5) in 5% KOH, germ pore small and indistinct, 1–2 guttulate, inamyloid. Basidia 18–23 × 7.0–8.0 μm, 4-spored, clavate, hyaline. Pleurocystidia 42–68 × 13–17 μm, thin-walled, fusiform, apex obtuse to subacute, rarely cystiform with a short rostrum, with one or two large internal oily drops, oily drops colourless and distinct or indistinct in 5% KOH, glassy-yellow (5B6–5B7) and very distinct in Melzer’s Reagent. Cheilocystidia scanty, 33–48 × 10–15 μm, similar to pleurocystidia, few and scattered, mix with pyriform or clavate, 21–39 × 11–13 μm-sized cells. Trama of gills consisting of parallel hyphae. Pileipellis a 2–3 cells deep layer of subglobose or pyriform cells which are 18–32 μm wide. Veil composed of 5.4–8.4 μm-broad hyphae, thin-walled and fawn (5A2-A3) hyphae in 5% KOH. Clamps rare, but observed in trama of gills, hyphae of stipe and at the base of the basidia and cystidia.

**Figure 3.**
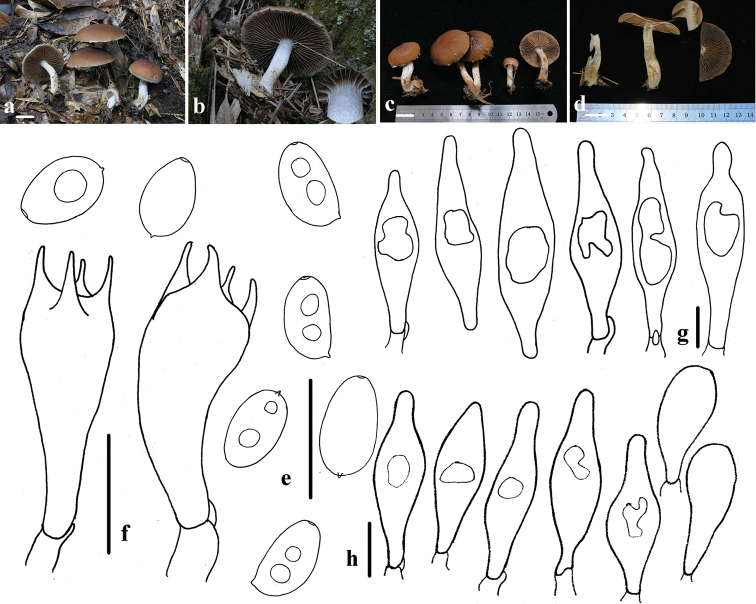
Basidiomata and microscopic features of *Typhrasa rugocephala***a–d** basidiomata **e** basidiospores **f** basidia **g** pleurocystidia **h** cheilocystidia. Scale bars: 20 mm (**a–d**); 10 μm (**e–h**).

##### Ecology and distribution.

Saprotrophic, solitary or gregarious on soil or humus in broad-leaved forests.

##### Other specimens examined.

China. Baishanzu National Nature Reserve, Zhejiang Province, 24 June 2020, Ya-Ping Hu, HFJAU1476; 28 June 2020, Sheng-Nan Wang, HFJAU1455, HMJAU58462.

## Discussion

*Typhrasa* was established by Örstadius et al. (2015), based on the main characters of having rostrate, hymenial cystidia with oily drops. Only *T. gossypina* and *T. nanispora* Örstadius, Hauskn. & E. Larss. were reported in that study. *T. gossypina*, as the type species of the genus, was, therefore, separated from *Psathyrella* (Fr.) Quél. This species can be separated from the two new species through its longer spores up to 9.0 μm long, 5.0–6.0 μm broad in front view and pleurocystidia often with a long rostrum (Kits van Waveren 1985; Örstadius et al. 2015). *T. nanispora* has smaller spores, 5.0–6.0 × 3.0–4.0 μm and can be thereby easily be distinguished (Örstadius et al. 2015). *T. rugocephala* is very easily confused with *T. polycystis*, but the former has two types of long gills arranged at intervals, scanty cheilocystidia and rarely rostrum can be found in fusiform pleurocystidia. In addition, *P. subtenacipes* are also reported to have oily drops in their cystidia (Smith 1972) and seems to be a candidate for *Typhrasa*, but study of type material is needed to settle this question. Morphologically, the spores of *P. subtenacipes* are up to 7.8–9.5 × 5.0–5.6 μm, significantly larger than the two new species (Smith et al. 1950; Smith 1972). A key to these related species is presented below:

### Key to related species

**Table:** 

1	Spores up to 9.5 μm long, 5.0–5.6 μm broad	***P. subtenacipes***
–	Not as above	**2**
2	Spores less than 6.0 μm long	***T. nanispora***
–	Spores over 6.0 μm long	**3**
3	Spores 7.0–9.0 μm long, 5.0–6.0 μm broad in front view	***T. gossypina***
–	Spores smaller, less than 8.0 μm long and 5.0 μm broad in front view	**4**
4	Long gills have two types concurrently: adnate to slightly decurrent and emarginate-adnexed, fusiform, apex obtuse to subacute, rarely cystiform with a short rostrum, cheilocystidia scanty	***T. rugocephala***
–	Gills adnexed, pleurocystidia variously-shaped, often fusiform and lageniform with rostrum	***T. polycystis***

## Supplementary Material

XML Treatment for Typhrasa polycystis

XML Treatment for Typhrasa rugocephala

## Supplementary materials

Supplementary material 1 Figure S1. Collection site of *Typhrasa rugocephala* and *T. polycystis* Data type image https://binary.pensoft.net/file/535263This dataset is made available under the Open Database License (http://opendatacommons.org/licenses/odbl/1.0/). The Open Database License (ODbL) is a license agreement intended to allow users to freely share, modify, and use this Dataset while maintaining this same freedom for others, provided that the original source and author(s) are credited.Sheng-Nan Wang, Ya-Ping Hu, Jun-Liang Chen, Liang-Liang Qi, Hui Zeng, Hui Ding, Guang-Hua Huo, Lin-Ping Zhang, Fu-Sheng Chen, Jun-Qing Yan
