# Supplementary material for: First record of the rare genus Typhrasa (Psathyrellaceae, Agaricales) from China with description of two new species
Source: MycoKeys. 2021 Apr 23;79:119–28. doi: 10.3897/mycokeys.79.63700 (PMC8087613; doi:10.3897/mycokeys.79.63700)
Supplement: Supplementary material 1 — Figure S1. Collection site of Typhrasa rugocephala and T. polycystis [file mycokeys-79-119-s001.docx]

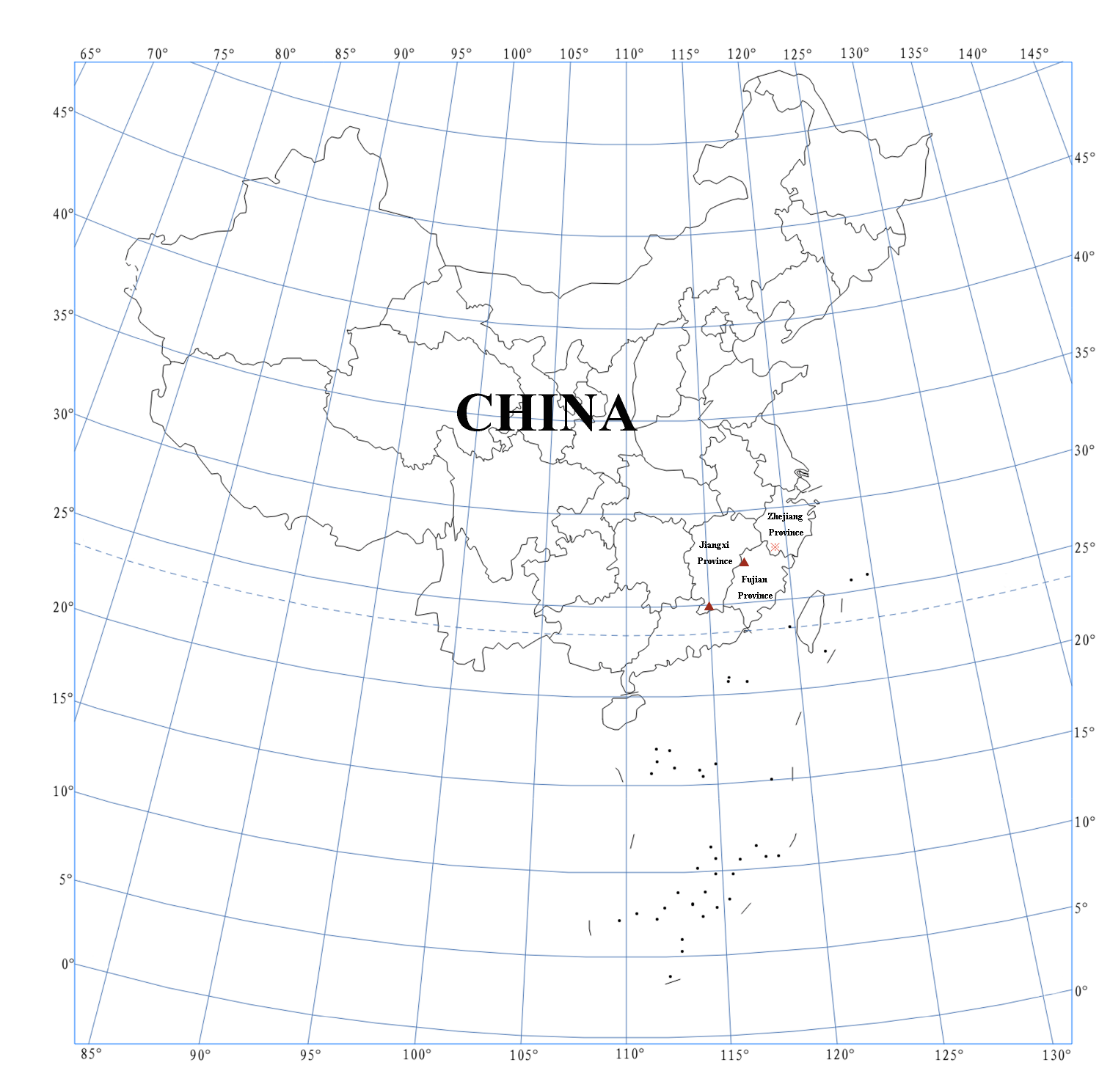
 Supplementary Fig. S1− collection site of two new species, ※: collection site of *Typhrasa rugocephala* ▲: collection site of *T. polycystis*.
